# Gastroesophageal reflux symptoms among Italian university students: epidemiology and dietary correlates using automatically recorded transactions

**DOI:** 10.1186/s12876-018-0832-9

**Published:** 2018-07-17

**Authors:** Irene Martinucci, Michela Natilli, Valentina Lorenzoni, Luca Pappalardo, Anna Monreale, Giuseppe Turchetti, Dino Pedreschi, Santino Marchi, Roberto Barale, Nicola de Bortoli

**Affiliations:** 1 Division of Gastroenterology–Versilia Hospital, Lido di Camaiore–Lucca, Italy; 2 Department of Computer Science–University of Pisa, Pisa, Italy; 3 Department of Biology–University of Pisa, Pisa, Italy; 4 Institute of Management, Scuola Superiore Sant’Anna of Pisa, Pisa, Italy; 5 Institute of Information Science and Technologies ISTI - Italian National Research Council (CNR), Pisa, Italy; 6 Division of Gastroenterology, Department of Translational Research and New Technologies in Medicine and Surgery–University of Pisa, Pisa, Italy

**Keywords:** Gastroesophageal reflux disease, GERD, Heartburn, Regurgitation, Diet, Prevalence, University students

## Abstract

**Background:**

Gastroesophageal reflux disease (GERD) is one of the most common gastrointestinal disorders worldwide, with relevant impact on the quality of life and health care costs.The aim of our study is to assess the prevalence of GERD based on self-reported symptoms among university students in central Italy. The secondary aim is to evaluate lifestyle correlates, particularly eating habits, in GERD students using automatically recorded transactions through cashiers at university canteen.

**Methods:**

A web-survey was created and launched through an app, ad-hoc developed for an interactive exchange of information with students, including anthropometric data and lifestyle habits. Moreover, the web-survey allowed users a self-diagnosis of GERD through a simple questionnaire. As regard eating habits, detailed collection of meals consumed, including number and type of dishes, were automatically recorded through cashiers at the university canteen equipped with an automatic registration system.

**Results:**

We collected 3012 questionnaires. A total of 792 students (26.2% of the respondents) reported typical GERD symptoms occurring at least weekly. Female sex was more prevalent than male sex. In the set of students with GERD, the percentage of smokers was higher, and our results showed that when BMI tends to higher values the percentage of students with GERD tends to increase. When evaluating correlates with diet, we found, among all users, a lower frequency of legumes choice in GERD students and, among frequent users, a lower frequency of choice of pasta and rice in GERD students.

**Discussion:**

The results of our study are in line with the values reported in the literature. Nowadays, GERD is a common problem in our communities, and can potentially lead to serious medical complications; the economic burden involved in the diagnostic and therapeutic management of the disease has a relevant impact on healthcare costs.

**Conclusions:**

To our knowledge, this is the first study evaluating the prevalence of typical GERD–related symptoms in a young population of University students in Italy. Considering the young age of enrolled subjects, our prevalence rate, relatively high compared to the usual estimates, could represent a further negative factor for the future economic sustainability of the healthcare system.

**Electronic supplementary material:**

The online version of this article (10.1186/s12876-018-0832-9) contains supplementary material, which is available to authorized users.

## Background

Gastroesophageal reflux disease (GERD) is a chronic condition which develops when the reflux of gastric contents into the esophagus causes troublesome symptoms and/or complications [1, 2]. It is well known that a wide and varying range of symptoms is associated with GERD. The two most typical symptoms are heartburn and regurgitation; however, patients may complain also other symptoms including chest pain, as well as extra esophageal manifestations such as chronic cough, asthma, and laryngitis [1]. GERD is one of the most common gastrointestinal disorders worldwide, with relevant impact on the quality of life and health care costs [3]. According to population–based studies, when defined as at least weekly heartburn and/or regurgitation, prevalence estimates generally range from 10 to 30% in Western populations, and only East Asia shows estimates consistently lower than 10% [4]. In addition, it is worth noting that evidence suggests an increase in disease prevalence since 1995 [4–7].

The etiology of GERD is largely unknown, and its pathogenesis is multifactorial in nature, mainly involving dysfunctions of the esophagogastric junction, ineffective esophageal acid and bolus clearance, increased intragastric pressure, and esophageal hypersensitivity [8, 9]. Moreover, the main established risk factors of GERD are heredity, obesity and tobacco smoking. Thus, according to a recent systematic review and current guidelines, the first step in GERD management consists of lifestyle interventions, such as weight loss, tobacco smoking cessation, avoiding late evening meals and head–of–the–bed elevation [10–13]. Along the same line, dietary modifications have also been proposed as first–line therapy for patients with GERD [14]. However, few and heterogeneous randomized clinical trials and observational studies have shown inconsistent or conflicting results about the putative role of specific dietary items in development of reflux symptoms [10, 15, 16]. In this context, the effect of the overall dietary pattern of a population on the risk of GERD has been scarcely evaluated. Recently, a cross–sectional study by Mone et al.[17] highlighted a beneficial effect of a Mediterranean diet in the occurrence of GERD.

Based on the above considerations, the aim of our study is to assess the prevalence of GERD based on self–reported symptoms among university students in central Italy. The secondary aim is to evaluate lifestyle correlates, particularly eating habits, in GERD students using data from a web survey and transactions automatically recorded through cashiers at university canteen.

## Methods

This work is part of a larger study aiming at studying the nutritional habits of University of Pisa students and the relation between these habits and gastrointestinal disorders. The project consists of several phases covering innovative aspects in terms of research and application of the results.

### Design and study population

In order to evaluate the correlation between eating habits and gastrointestinal disturbances, a web–survey was created and launched through an app, ad hoc developed for an interactive exchange of information with students. Participants were recruited among students of University of Pisa and participation to the survey was voluntary. To ensure adequate widespread of the project among the target population, information was diffused through various means: the web portal of University of Pisa, the email contact list of University of Pisa and brochures into the canteen area. The students were informed that they would have been notified on the results of their questionnaire and that a number of dietary recommendations suitable for any detected disorder would have been available. Students can access the survey only through the university’s personal credentials and all individuals who agreed to participate gave an informed consent through the web–app.

The survey was built (in Java) on *Liferay Community Edition*, a Content Management System (CMS) that can create the succession of questions and possible answers with images and scores.

The survey included both qualitative and quantitative questions devoted to the collection of anthropometric data and lifestyle habits of the student (i.e., height, weight, smoking, coffee consumption), as well as possible gastrointestinal symptoms.

### Data sources and data collection

To reach the project’s goals, three different sources of data were used and merged to obtain the database for the analysis:

- data from the University administrative archive;
- data collected with a web–app that allows users a self–diagnosis for the gastrointestinal disturbances through a simple questionnaire;
- data on meals consumption provided by the regional company that manages the University canteen.

The University archive, used to identify the overall students enrolled in the academic year 2016–17, provides demographic and academic career data for all students enrolled.

For those students participating into the web–survey and/or using the university canteen additional information was added as detailed below.

The web questionnaire comprises specific sections to investigate the presence of gastrointestinal disorders, with particular reference to GERD, and a general section devoted to the collection of demographic and anthropometric data (weight and height), daily coffee consumption and the current smoking behavior.

Since one of the main attributes involved in the study is the Body Mass Index (BMI), we calculated it as the weight in kilograms divided by the square of the height in meters (*kg*/*m*^2^), and, accordingly with WHO definition the class where defined as show in Table 1. Questions on how many cups of coffee were consumed by students and a question in which we ask whether the student is a smoker or a non-smoker were included.

**Table 1:** The International Classification of adult underweight, overweight and obesity according to BMI

Classification	*kg*/*m*^2^
Underweight	<18.50
Normal range	18.50−24.99
Overweight	25.00−29.99
Obesity class I	30.00−34.99
Obesity class II	35.00−39.99
Extreme obesity	≥40.00

Questions related to the assessment of the GERD were formulated on the basis of a simplified version of a validated questionnaire, the GERDq questionnaire, limiting the questions only to typical reflux symptoms. [1, 18–20].

In the questionnaire, heartburn was defined as a burning sensation or pain behind the breast bone in the chest, and regurgitation was defined as the perception of backflow of gastric content coming into the throat or mouth. In particular, the frequency and the intensity of typical GERD-related symptoms were evaluated with a Likert scale and a visual analog scale (VAS).

The survey was launched on October 2016 and it is still online. The data used for the present work cover a period of 200 days, from the 21^*st*^ of October 2016 to the 10^*th*^ of May 2017.

The “*Azienda Regionale per il Diritto allo Studio Universitario*” (DSU), a public corporation operating throughout the Region in order to offer food services to students as well as accommodation, grants or scholarships to the most deserving ones, administered the three University canteens students. Thanks to the personal magnetic cards used to access the canteen, the DSU owns a dedicated database collecting, for each transaction (i.e. a meal), information about date and hours, type of meal, student ID and price applied. Number and type of dishes are also automatically recorded for meals consumed in one of the canteens equipped with an automatic system for the detailed collection of meals consumed. That source of data was used in the present analysis to extract records about meal consumption for those students accessing the canteen and participating into the web survey.

### Statistical analysis

A Heckman–type selection model [21] was used to assess the presence of selection bias in the sample of students answering the survey, to determine whether the GERD prevalence estimated among University students could be obtained directly from the observed data, or whether there is need of a correction for selection bias. Sample selection occurs when the data at hand are not a random sample from the population of interest. To deal with that, Heckman—type selection models have been widely used in economics and social science to evaluate whether the mechanism determining the participation (selection) into a survey is independent of the presence of the outcome of interest. The selection model typically consists in a bivariate regression comprising a selection equation, which describes survey participation, and an outcome equation, predicting the outcome of interest. The two equations are linked through a correlation parameter, *ρ*, representing the covariance between the outcome and the participation conditional on observed covariates. A statistically significant value of *ρ* implies a relation between the process of participation into the study and the outcome, thus providing evidence about the presence of selection bias and hence the need to correct estimates coming from the observed data. A negative estimate of *ρ* means that subjects in which the outcome is present are less prone to participate, vice versa in case of a positive value.

A bivariate probit Heckman–type selection model was used in the present analysis. Age and gender were used as covariates in the selection equation, while, to comply with exclusion restriction, only gender was used as covariate in the outcome equation.

Different statistical analysis were performed: descriptive statistics on the data collected, a study of the GERD prevalence and lifestyle correlates with both a monovariate and a multivariate approach (logistic regression). The independent variables used in the model are: Gender (Female), a dummy variable that is equal to 1 when the student is a female; Smoking habit (Smoker): dummy variable that is equal to 1 when the student is a smoker; Number of coffee, continuous variable; BMI Underweight, dummy variable; BMI Overweight, dummy variable; BMI Obesity^1^, dummy variable.

Eating habits of students were analysed and compared based on food items automatically recorded from cashiers’ transactions at University canteens. All food was grouped into 12 specific groups on the basis of the main dish and the type of cooking: pasta and rice, soups, legumes, vegetables and salad, potatoes, meat, seafood, fruits, sweets, fried food, sandwiches and pizza, pies and omelette. The frequency of different food groups consumption was described in terms of the number of times the specific group is selected over the total number of accesses and reported as percentage. The latter analysis was performed on all users and also on the subgroups of frequent users. Frequent users were defines as those students accessing the canteens at least 20 times in the year on the basis of the 75^*th*^ percentile of the distribution of the number of yearly accesses.

Continuous variables were described in terms of median [ 25−75^*th*^ percentile] and Mann-Whitney U test was used to perform comparisons between groups. Categorical variables are presented as number of subjects and percentage and Fisher or *χ*^2^ tests were used to compare the distribution of variables between groups.

All analyses were performed using *Python*, STATA and SPSS.

## Results

During the 200 days the survey was online, 3012 questionnaires were collected, about 6.3% of the students enrolled in the University of Pisa. In Table 2 the details of the respondents.

**Table 2:** Descriptive characteristics of the respondents to the web-questionnaire

	n	%
*Gender*		
Male	1136	37.6
Female	1884	62.4
*BMI class*		
Underweight	300	9.9
Normalweight	2268	75.1
Overweight	366	12.1
Class I obesity	65	2.2
Class II obesity	15	0.5
Extreme obesity	6	0.2
*Age by gender*		
	F	M
Median	24.0	24.0
Mean	26.7	25.9
Q1	22.0	22.0
Q3	27.0	27.0
IQR	5.0	5.0

62.4% of the respondents (*n*=1,884) were females and the remaining 37.6% (1,136) were males. The median age of respondents was 23 years (with a standard deviation of 3.6 years and an interquartile range of 5 years). Table 3 shows the distribution of respondents by gender and Body Mass Index (BMI): while the “Normal Weight” is the mode of the distribution, we observe that males have a tendency toward overweight, while females have a tendency toward underweight (statistically significant differences, with *P*-value <0.00001).

**Table 3:** Respondent to the web-questionnaire by gender, BMI and coffee consumption

	F	M	Female (%)	Male (%)
*BMI class * ^∗^				
Underweight	256	44	13.6	3.9
Normal weight	1422	846	75.5	74.5
Overweight	163	203	8.7	17.9
Class I obesity	33	32	1.8	2.8
Class II obesity	5	10	0.3	0.9
Extreme obesity	5	1	0.3	0.1
*Number of coffee consumed * ^∗^				
None	439	301	23.3	26.5
1–2	966	539	51.3	47.4
3–4	437	256	23.2	22.5
5 +	42	40	2.2	3.5
*Smoking habits*				
Yes	370	226	19.6	19.9
No	1514	910	80.4	80.1

^*^P-value <0.05 for comparison between males and females distribution of the variables

The original numerical variable “number of coffee” was categorized for descriptive purposes

Regarding coffee consumption, there is a statistically significant difference in the coffee consumption (see Table 3), with a higher number of coffees per day for the female population (*P*-value=0.023). Furthermore we observe that for both males and females almost 20% of the students are smokers, with no statistically significant differences among gender.

### GERD prevalence and lifestyle correlates

Based on the available evidence and the bivariate probit Heckman–type selection model specification, estimate of *ρ* was not significantly different from zero (Table 4). This means that the hypothesis of the absence of selection bias could not be rejected, accordingly prevalence estimates and correlates were derived directly from the observed data.

**Table 4:** Estimates from the Heckman–type selection model

	Coef.	Std. Err.	*P*-value	95% CI
Outcome model: Presence of GERD				
Gender	0.255	0.051	<0.001	(-0.354;-0.156)
(cons)	1.302	0.448	0.004	(-2.180;-0.424)
Selection model: Survey Participation				
Gender	0.222	0.018	<0.001	(-0.257;-0.187)
Age	0.012	0.002	<0.001	(-0.015;-0.009)
(cons)	0.878	0.047	<0.001	(-0.971;-0.786)
*ρ*	0.582	0.316		(-0.263;0.922)

**Fig. 1:**
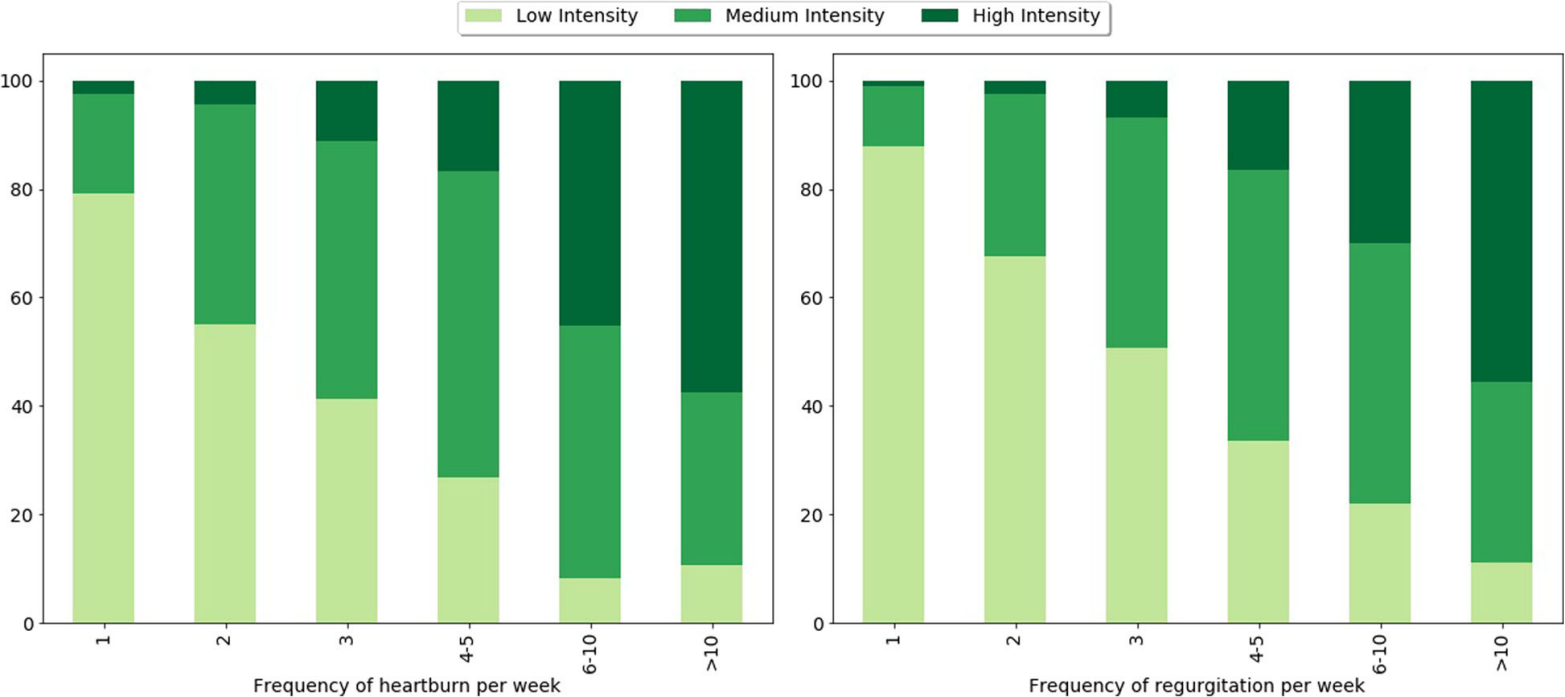
Intensity of heartburn and regurgitation symptoms by their frequency

Table 5 shows the distribution of students’ characteristics and lifestyle habits according to the presence of GERD. 792 students out of 3020 respondents (26.2%) reported typical GERD symptoms occurring at least weekly. In particular, heartburn and regurgitation were reported by 47 and 53% of the students, respectively. As reported in Fig. 1, most reflux symptoms which were experienced only once a week were very mild in severity, and the severity increases with the increase of the weekly frequency of the symptoms.

**Table 5:** Prevalence of GERD by BMI class, Smoking habits and number of coffee consumed

	No GERD		GERD	
	n	%	n	%
*BMI class * ^∗^				
Underweight	216	9.7	84	10.6
Normal weight	1694	76.0	574	72.5
Overweight	265	11.9	101	12.8
Class I obesity	42	1.9	23	2.9
Class II obesity or more	11	0.5	10	1.3
*Smoking habit * ^∗^				
Non smoker	1827	82.0	597	75.3
Smoker	401	18.0	195	24.7
*Number of coffee consumed * ^∗^				
None	556	25.0	184	23.2
1–2	1131	50.8	374	47.2
3–4	483	21.7	210	26.5
5 or more	58	2.6	24	3.0

^*^P-value <0.05 for comparison in GERD prevalence among groups

Female students are more affected by the symptoms, 28.2% versus 22.9%, with this difference statistically significant (*P*-value =0.0014).

Furthermore, a cross tabulation between GERD and BMI was performed. In Table 5 the distribution of the two variables.

Since the number of students in Class II obesity and Extreme obesity are low they were merged in one category to have a more precise idea of this distribution. As expected, overweight and obese were more frequent among GERD (*P*-value = 0.030). On the other side, in the set of students with GERD, the percentage of smokers is higher than the percentage of students without GERD (*P*-value <0.001), and also coffee consumption play an important role in the prevalence of GERD (*P*-value = 0.036).

Same results can be drawn looking at the odds ratios (Table 6) of these correlates: smoking habits and overweight/obesity have a positive impact on the GERD prevalence. Being a female student also seems to be related to GERD prevalence. These conclusions are based on univariate analysis, but for having a more informative results a multivariate logistic regression analysis was performed on the data with the aim of explaining the relationship between the dependent binary variable (GERD) and all possible independent variables together.

**Table 6:** Odds ratio and confidence interval for lifestyle correlates with monovariate and multivariate analysis

	Monovariate				Multivariate			
		Conf. Interval				Conf. Interval		
	Oddsratio	[0.025	0.975]	*Sig.*	OddsRatio	[0.025	0.975]	*Sig.*
Gender(F)	1.45	1.19	1.77	*0.00*	1.52	1.24	1.87	*0.00*
Smoker(Yes)	1.71	1.35	2.17	*0.00*	1.6	1.25	2.05	*0.00*
Number of coffee	1.1	1.03	1.18	*0.01*	1.04	0.97	1.12	*0.25*
BMI Underweight	1	0.72	1.39	*0.99*	0.97	0.69	1.37	*0.88*
BMI Overweight	1.17	0.89	1.55	*0.27*	1.26	0.94	1.69	*0.12*
BMI Obesity	2.04	1.19	3.49	*0.009*	1.97	1.14	3.43	*0.02*
Intercept					0.23			*0.000*

As shown in Table 6, the variables Gender(F), Smoker(Yes) and BMI Class Obesity (aggregation of the 3 different levels of obesity) are highly statistically significant: smokers are more likely to experience GERD symptoms, such as female students. The students in the Obesity class have an odd ratio greater than one, which means that Obesity can be considered a determinant of GERD symptoms. The coffee consumption seems to have a positive impact on GERD presence.

### Dietary pattern in GERD

Overall, 32.5% (980) of all respondents accessed at least once of the University canteen in the academic year 2016–17, and about 13.1% (395) were frequent users. The prevalence of GERD among the subgroup of respondents accessing the canteen was 25.9%, not statistically different from the value in the overall group of respondents (p=0.882). Characteristics and lifestyle of overall users are reported in Additional file 1: Table S1.

When evaluating food choices, among all users a lower frequency of legumes choice was observed in GERD students (*P*-value = 0.002) while, when considering frequent users we find a significant (*P*-value =0.034) lower frequency of choice of pasta and rice in GERD students (see Table 7).

**Table 7:** Frequency (as percentage) of food group choice overall and by presence of GERD, among all users and the subgroup of frequent users

All users			
	Overall (*n*=980)	No GERD (*n*=726)	GERD (*n*=254)
Sweets	0 [0-10.1]	0 [0-10.4]	0 [0-10]
Fruits	40 [10.1-73.3]	40.4[11.1-72.6]	40[8.2-75]
Vegetables and salad	36.8 [14.3-59]	36.9 [15.5-60]	36.2[10.3-57.3]
Soups	0 [0-6.5]	0 [0-6.3]	0 [0-7.1]
Seafood	0 [0-6.3]	0 [0-6.3]	0 [0-6.8]
Legumes ^∗^	0 [0-8.2]	1.1 [0-9]	0 [0-6.1]
Sandwiches and pizza	8.3 [0-27.7]	7.1 [0-26.7]	10 [0-33.3]
Meat	27.3[1-50]	27.8 [3.6-50]	25 [0-47.7]
Fried foods	20 [0-36.8]	20 [0-36.4]	28.9[0-37.7]
Pies and omelette	3.9 [0-11.8]	4.4 [0-11.8]	2.3 [0-12.1]
Pasta and rice	64.3 [33.3-90.6]	65.8 [33.3-89.9]	55.7 [30.1-93.6]
Potatoes	14 [0-25]	14.3 [0-25]	13.2 [0-25]
Frequent users			
	Overall (*n*=395)	No GERD (*n*=304)	GERD (*n*=91)
Sweets	5.15[1.01-12.5]	5.4[0.9-12.5]	4.8[1.5-12.5]
Fruits	44.3[22.1-70.0]	44.4[21.8-70]	40.4[22.8-67.7]
Vegetables and salad	37.3[24.5-53.2]	37.7[26.2-54.7]	35.7[17.8-50]
Soups	3.1[0-10]	3.1[0-10.1]	2.9[0-9.7]
Seafood	3.6[0-8.3]	3.6[0.8-8.3]	2.7[0-8.3]
Legumes	4.3[1.2-7.9]	4.4[1.46-8.3]	3.4[0-6.8]
Sandwiches and pizza	14.0[5.7-27.9]	13.6[5.4-26.8]	17.9[8.4-32.6]
Meat	30.7[17.2-43.0]	31.5[17.3-43.0]	29.7[17.1-44.1]
Fried foods	28.4[17.4-39.3]	28.57[17.7-39.1]	27.3[16.7-40]
Pies and omelette	7.5[4.05-12.5]	7.3[4.2-12.0]	7.7[3.5-14.1]
Pasta and rice ^∗^	62.5[40.2-80]	65.5[40.9-81.1]	54.8[35.2-75.7]
Potatoes	17.1[11.5-24.3]	17.2[12.0-24.5]	17.1[11.3-23.8]

^*^P-value <0.05 for comparison between groups

No differences among students with and without GERD were found also when distinguishing male and female, or by distinguishing students by BMI category (i.e., normal weight and overweight/obese) (see Additional file 1: Table S3-S5).

## Discussion

This is the first study evaluating the prevalence of typical GERD-related symptoms in a young population of University students in Italy. Our data show a prevalence rate of weekly symptoms in 26.2% of the total respondents. In a previous population-based study performed in Italy, 700 employers in Pavia answered a physician interview about typical GERD-related symptoms. The results showed a prevalence of heartburn and regurgitation of 7.7 and 6.6%, respectively [22]. On the other hand, in European population-based studies,the range of GERD prevalence estimates (defined as at least weekly heartburn and/or regurgitation) was 8.8%–25.9% [5]. Since systematic reviews suggested that the prevalence of GERD is increasing [4, 5], the results of our study are in line with the values reported in the literature. Nowadays, GERD is a common problem in our communities, and can potentially lead to serious medical complications (i.e. Barrett’s esophagus, esophageal adenocarcinoma); moreover,the economic burden involved in the diagnostic and therapeutic management of the disease has a relevant impact on healthcare costs. In this context, considering the young age of enrolled subjects, our prevalence rate, relatively high compared to the usual estimates, could represent a further negative factor for the future economic sustainability of the healthcare system.

While to analyze GERD risk factors it was not the primary goal of this article, it is worth noting that female sex was more prevalent than male sex. Moreover, in the set of students with GERD the percentage of smokers was higher. However, current evidence of associations of GERD with sex or smoking habits are conflicting and inconclusive. On the other hand, evidence shows incontrovertibly a strong association between overweight/obesity and occurrence of GERD symptoms and its complications, both analyzing data with a monovariate approach or a multivariate approach,so that the global rising prevalence of GERD seems to be related to the rapidly increasing prevalence of obesity, which has occurred in the last few decades [23–27]. Moreover, much evidence indicates the effectiveness of weight reduction on symptom relief, at least in GERD patients who are overweight or obese [28–30]. In line with these assumptions, our results show that when BMI tends to higher values the percentage of students with GERD tends to increase.

Our study has several limitations. First of all, the observational nature of the study and the groups included in the analyses that are represented by students using the canteen and those participating into the survey thus precluding the generalizability of results. The aforementioned limitations were considered when performing statistical analyses and while results from the Heckman–type selection model suggested no presence of selection bias, caution is needed in interpreting results from that model. As only few variables were available for the inclusion as covariates, the model adapted could fail in explaining the mechanism underling survey participation. It is well known that [31] the choice of selection variables can impact model estimates, thus despite significance of the variables used in the present analysis, poor fitting of the model suggested that other variables than those collected in the present study may impact on the survey participation. In particular, psychological factors, as attitudes toward the compliance with survey study and sensitivity to the theme investigated, probably play a key role in determining survey participation and should be addressed in future studies. Anyway, to support findings from the present study, hypothesizing that possible differences and attitudes among students could be reflected in the type of studies they attended, verification of the presence of selection bias was also performed in the subgroups of students using the University canteen for which other variables were available. Including also University Department as covariate in the selection model did not improve fitting and results remained invariant, still indicating no presence of selection bias (data not shown). Moreover, prevalence estimate obtained from our analysis is coherent with available evidence.

The second limitation of this study is related to the intrinsic difficulty of identifying which patients actually suffer from GERD. Indeed, it is well known that patients with GERD may present with atypical symptoms, such as cough, asthma, laryngitis, in the absence of typical esophageal symptoms, and other patients with GERD experience no symptoms at all [1, 32]. As a consequence, our estimates will include some students who have typical GERD symptoms generated by mechanisms other than reflux (i.e., functional heartburn), as well as will not include students whose symptoms are sporadic or absent. However, such limitation represents a common problem for all population-based studies aimed at investigating GERD prevalence through questionnaires [5]. Indeed, our prevalence estimate is coherent with available evidence. In this context, it must be underlined that we used a simplified version of the GERDq questionnaire to assess the presence of GERD, limiting the questions only to typical reflux symptoms.

The third limitation of our study is that this is an observational study in a very selected group of people (i.e., students eating at the canteen) whose results cannot be directly applied to the general population.

According to our secondary aim, we explored the possible association between different dietary patterns and GERD to assess the effects of diet on disease risk. In this regard, it is worth noting that a strength of this article is that food items are automatically recorded from cashiers’ transactions at University canteen. Thus, these data are not affected by the well-known problem of memory effect [33], or by the bias due to the tendency in survey respondents to answer questions in a way that will be viewed favourably by others, the “social desirability” bias [34]. Of note, when evaluating food choices, among all users a lower frequency of legumes choice was observed in GERD students. However, this data was not confirmed when considering food choices among frequent users. On the other hand, when considering frequent users we found a significant lower frequency of choice of pasta and rice in GERD students. On the basis of the available literature and of the known pathophysiological mechanisms of GERD, it is not currently possible to speculate on why this low frequency of consumption and how this can affect the symptoms[35–40]. For example, some studies have shown that consumption of a high-fat diet was associated with GERD, but several other studies reported conflicting data. El-Serag et al. have demonstrated that fruits, vegetables, and high-fiber diets are inversely associated with GERD [35], whereas Zheng et al. found that none of these items was associated with the risk of GERD symptoms [39]. Furthermore, high-fat foods and chocolate are empirically indicated as foods able to reduce lower esophageal sphincter pressure or to prolong gastric emptying. Recently, an interesting study by Keshteli et al. [41] found that higher dietary glycaemic index and glycaemic load may be risk factors for uninvestigated heartburn and uninvestigated chronic dyspepsia in men, as well as normal-weight subjects, but not in women and overweight individuals. Thus, consuming a low-glycaemic index diet might be beneficial in normal-weight patients with uninvestigated heartburn. Obviously, these findings warrant evaluation in prospective studies to establish the potential role of carbohydrate quality in the management of GERD. However, there have been no cessation trials evaluating the impact on GERD outcomes. However, our findings show an interesting aspect to be kept in careful consideration and to be reassessed, continuing to acquire objective data on the choice of food over time. Overall, our results showed no differences among students with and without GERD when distinguishing male and female, or by aggregating students by BMI category. A major concern of this analysis is that the dietary intake information provided by DSU might be not reflective of participant’s habitual dietary intake. Indeed, we do not have a complete mapping of what each student eats every day, outside the University canteen. However, this study might be considered as pilot study to properly assess the possible influence of different dietary patterns on gastrointestinal diseases based on real and objective data about eating habits. Moreover, as regards GERD, according to current available literature, the effectiveness of dietary recommendations has not been shown, and, thus, recommendations are to have a generally healthy diet and to avoid food items that, in the experience of the patient, trigger symptoms [23].

## Conclusion

To our knowledge, this is the first study evaluating the prevalence of typical GERD-related symptoms in a young population of University students in Italy.Our data show a prevalence rate of weekly symptoms of 26.2%. Considering the young age of enrolled subjects, our prevalence rate, relatively high compared to the usual estimates, could represent a further negative factor for the future economic sustainability of the healthcare system.

When assessing the effects of diet on disease risk, we found, among all users, a lower frequency of legumes choice in GERD students and, among frequent users, a lower frequency of choice of pasta and rice in GERD students. Since food items are automatically recorded from cashiers’ transactions at University canteen, this study might be considered as pilot study to properly assess the possible influence of different dietary patterns on gastrointestinal diseases based on real and objective data about eating habits.

## Additional file

Additional file 1 Supplementary tables. (TEX 11 kb)

## Abbreviations

BMI: Body mass index
CMS: Content management system
DSU: Azienda Regionale per il Diritto allo Studio Universitario
GERD: Gastroesophageal reflux disease
IQR: Inter quartile range

## Footnotes

1 The 3 levels of obesity were aggregated due to the reduced number of observations
